# Integration of a Mental Health App (e-MICHI) Into a Blended Treatment of Depression in Adolescents: Single-Group, Naturalistic Feasibility Trial

**DOI:** 10.2196/58427

**Published:** 2025-05-01

**Authors:** Lena Lincke, Tim Martin-Döring, Andrea Daunke, Antonia Sadkowiak, Daria Alexandra Nolkemper, Nina Sproeber-Kolb, Stefanie Bienioschek, Olaf Reis, Michael Kölch

**Affiliations:** 1 Department of Child and Adolescent Psychiatry, Neurology, Psychosomatics and Psychotherapy, Rostock University Medical Center, Gehlsheimer Str. 20, Rostock, 18147, Germany; 2 Partner Site Greifswald/Rostock, German Center for Child and Adolescent Health (DZKJ), Rostock, Germany; 3 Brandenburg Medical School Theodor Fontane (MHB), Neuruppin, Germany; 4 Department of Child and Adolescent Psychiatry, Psychotherapy and Psychosomatics, University Hospital Ruppin-Brandenburg (ukrb), Neuruppin, Germany; 5 School of Social Work, Baden-Wuerttemberg Cooperative State University, Stuttgart, Germany; 6 Faculty of Health Sciences, Joint Faculty of the University of Potsdam, the Brandenburg University of Technology Cottbus-Senftenberg and the Brandenburg Medical School Theodor Fontane, Potsdam, Germany

**Keywords:** adolescents, major depressive disorder (MDD), cognitive behavioral therapy (CBT), digital health interventions (DHIs), feasibility, adherence, outpatient setting, mobile application, accessibility, availability, efficacy, mobile phone

## Abstract

**Background:**

Major depressive disorder is a common diagnosis among adolescents. Cognitive behavioral therapy is recommended as the first line of treatment. Digital health interventions, such as apps, could contribute to treatment. Advantages could be easy accessibility and availability, reduced time for face-to-face therapy, and the ability to intensify therapy by incorporating it into the patients’ everyday lives. Challenges such as low adherence rates are common in digital health interventions. Therefore, they need to undergo rigorous testing for feasibility and effectiveness.

**Objective:**

An evaluated, cognitive behavioral therapy–based face-to-face therapy program for depression in adolescents was transformed into an app called e-MICHI. This study examined its feasibility and efficacy for use in blended therapy in outpatient settings.

**Methods:**

Adolescents aged 12 to 18 years with major depressive disorder receiving outpatient care were recruited from 2 university hospitals (n=36 included in analysis). The e-MICHI intervention combined daily app engagement over 6 weeks with 3 face-to-face sessions with a therapist. Feasibility was measured using various variables, including an adherence score (0=no or little patient engagement to 3=excellent engagement) and engagement rates (number of modules completed, number of messages sent by participants via the in-app messenger), satisfaction ratings from both participants and therapists, as well as participants’ ratings of the usefulness of the antidepressant strategies covered in the app and the transfer of these strategies to everyday practice. Trends of efficacy were evaluated from multiple perspectives (participant self-rating, independent rater, or therapist), using the Beck Depression Inventory-II, the Children’s Depression Rating Scale-Revised, and the Clinical Global Impressions-Severity Scale. Feasibility metrics were assessed by analyzing their central tendency and dispersion, efficacy data were analyzed using a repeated measures ANOVA.

**Results:**

e-MICHI was positively evaluated by both participants and therapists (participants: mean 7.3, SD 1.2 and therapists: mean 7.3, SD 1.1, on a scale from 0=bad to 10=excellent). Participants demonstrated high adherence rates (nearly 80%, n=25, received a “good” or “excellent” adherence score) and showed overall good engagement (app modules completed [maximum 6]: mean 5.03, SD 1.27 and messages sent via messenger: mean 23, SD 22.1). Participants rated the psychoeducational content of the app as particularly useful and reported consistent practice of the e-MICHI strategies in everyday life. Use of the app was associated with a significant reduction of depressive symptoms (before app use vs 3-month follow-up, Beck Depression Inventory-II: mean −6.76, SD 11.49, *P*=.01; Children’s Depression Rating Scale-Revised: mean −16.45, SD 16.76, *P*<.001; Clinical Global Impressions-Severity Scale: mean −1.1, SD 1.24, *P*<.001).

**Conclusions:**

While acknowledging its limitations, such as the small number of participants and the limited validity concerning efficacy, this study confirms the feasibility of e-MICHI for treating adolescent depression in outpatient settings.

## Introduction

Major depressive disorder (MDD) is a prevalent psychiatric diagnosis among adolescents and the primary cause of inpatient treatment of adolescents in Germany [1,2]. Cognitive behavioral therapy (CBT) is the first-line treatment [3,4].

Digital health interventions (DHIs) could contribute to facilitated access to psychotherapeutic treatment [5]. Adolescents and young adults show the greatest openness and acceptance toward mental e-health interventions [6]. However, there is a scarcity of e-health interventions for depression in adolescents. Furukawa et al [7] found that all forms of internet-based CBT were superior to a waiting list control condition. Similarly, meta-analytical evidence indicates that DHIs are moderately to considerably effective (standardized mean difference 0.16 to 0.76) when compared to primarily inactive control groups [8]. Numerous studies have reported high dropout rates and low adherence levels for nonguided e-health interventions [9,10]. DHIs that include some form of personalized support, for example, from psychotherapeutic professionals, seem to be more efficacious due to higher adherence rates [11]. In addition to adherence, transferability presents a further challenge. The transfer of the contents and skills learned in e-health interventions to everyday practice by users seems to be poor: within the SPARX-R program, an intervention for adolescent depression, only around 40% of users practiced few, if any, of the techniques taught in the program [12]. Incorporating engaging elements such as text, audiovisual aids, and gamification, alongside the real-world implementation and involvement of parents and professionals could enhance this transfer [13].

There is an increasing availability of various DHIs for adolescents [14]. Due to the lack of scientific evaluation, Christ et al [15] called for further research and long-term follow-up assessments of these interventions. Furthermore, Seiferth et al [13] established guidelines for evidence-based DHIs including individuals with suicidal thoughts, emphasizing robustness, comparability, crisis planning, and access to additional treatments. This study aimed to assess the feasibility and indicators for efficacy of a blended DHI application, based on an evaluated face-to-face therapy program for depression in adolescents. The target population was adolescents with clinically verified depressive symptoms who were receiving child and adolescent psychiatric and psychotherapeutic care.

## Methods

### Procedure

This study used a single-group design to investigate the feasibility of a 6-week blended DHI for adolescents with depression. To this end, treatment adherence and the effects on symptom severity during the intervention period and a 90-day follow-up were analyzed.

This study was conducted at the Departments of Child & Adolescent Psychiatry at Rostock University Medical Center and University Hospital Ruppin-Brandenburg, in association with Brandenburg Medical School, Neuruppin. Adolescents aged between 12 and 18 years who met the criteria for a MDD, according to the *ICD-10* (*International Statistical Classification of Diseases and Related Health Problems, 10th Revision*) [16] as diagnosed by a certified child and adolescent psychiatrist or psychotherapist and were receiving outpatient care were included in the trial. Patients who met these inclusion criteria were informed about this study and asked whether they wanted to participate. If they and their parents agreed to participate, MDD diagnoses were confirmed by independent raters using the Beck Depression Inventory-II (BDI-II) [17] and the Children’s Depression Rating Scale-Revised (CDRS-R) [18]. Additionally, a comprehensive diagnostic assessment was performed (Table 1).

**Table 1.** Overview of assessments conducted at different time points in a feasibility trial integrating the e-MICHI mental health app into a blended treatment of adolescent depression.

	Screening or recruitment	Pretreatment (T1)	Interim (T2)	Posttreatment (T3)	Follow-up (T4)
Trial days	–7	1	21	42	T3 +90
Diagnosis of depressive disorder	✓	N/A^a^	N/A	N/A	N/A
Inclusion and exclusion criteria	✓	N/A	N/A	N/A	N/A
Patient information and consent	✓	N/A	N/A	N/A	N/A
Demographics	N/A	✓	N/A	N/A	N/A
Previous and concomitant medication	N/A	✓	N/A	N/A	N/A
Concomitant diseases	N/A	✓	N/A	N/A	N/A
Treatment expectancy	N/A	✓	N/A	N/A	N/A
Adherence score	N/A	N/A	N/A	N/A	✓
Satisfaction and usability^b^	N/A	N/A	N/A	✓	N/A
BDI-II^c^	N/A	✓	✓	✓	✓
CDRS-R^d^	N/A	✓	N/A	✓	✓
CGI-S^e^	N/A	✓	✓	✓	✓
Adverse events	N/A	N/A	✓	✓	✓

a N/A: not applicable.

b Once per participant and once per therapist.

c BDI-II: Beck Depression Inventory-II [17].

d CDRS-R: Children’s Depression Rating Scale-Revised (clinician rating) [18].

e CGI-S: Clinical Global Impressions-Severity Scale [19].

Additional psychopharmacological antidepressant treatment was allowed, and treatment as usual was provided as needed, to ensure that this study’s population was representative of “real world” patients in routine care. Exclusion criteria for participation in the trial were acute suicidality requiring acute inpatient treatment, severe other psychiatric diagnoses such as schizophrenia and bipolar-disorder, and IQ below 85 or inadequate German language skills, as this would hinder the use of the program. Assessment for these criteria was based on clinical judgement following the Kinder-DIPS diagnostic interview for mental disorders [20].

### Intervention

**Figure 1.**
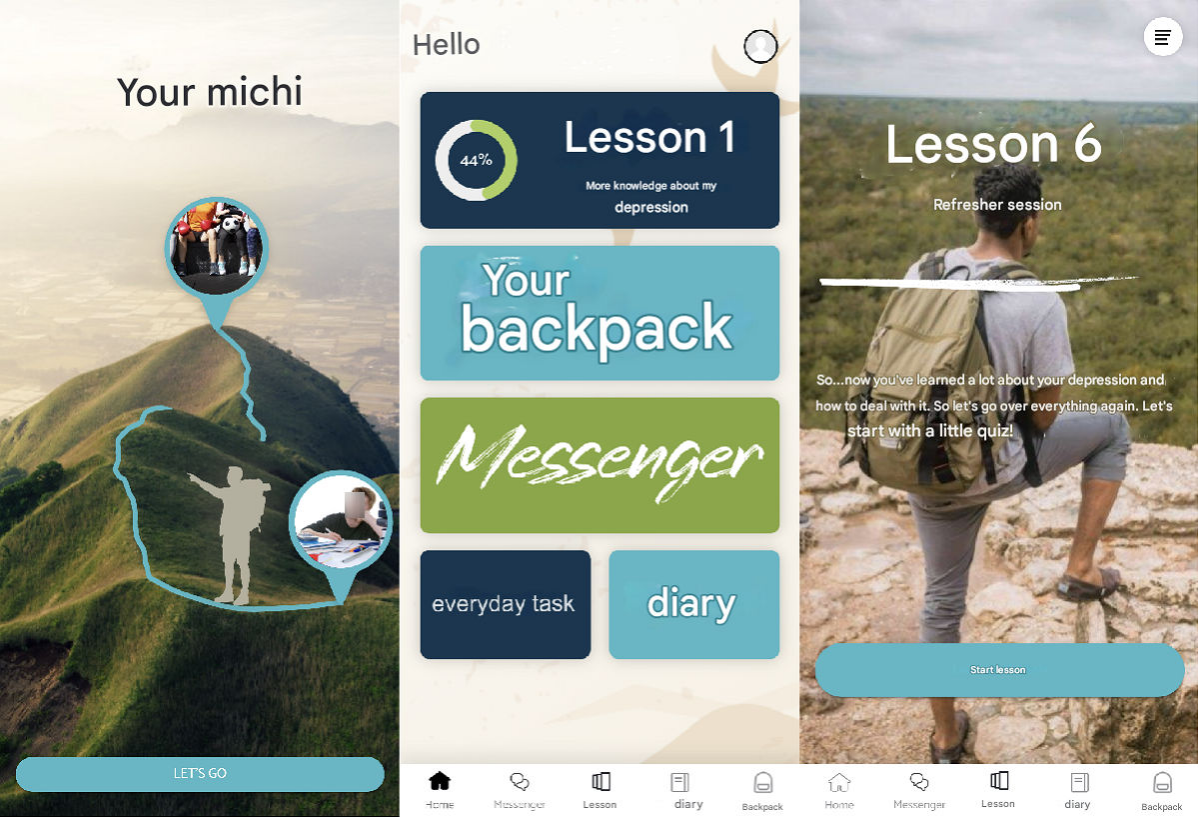
Screenshots of the e-MICHI app interface, illustrating the home screen (providing an overview of progress and tasks) and the module start screen (introducing structured activities and content).

The e-therapy was delivered through a newly developed app called “e-MICHI” (Figure 1). e-MICHI is based on the MICHI therapy program (“Manualized Intervention to Cope With Depressive Symptoms, Help Strengthen Resources and Improve Emotion Regulation”) [21], which has been shown to be effective for the treatment of depression in adolescents [22]. MICHI incorporates the proven effective elements of CBT for depression: psychoeducation, behavioral activation, cognitive restructuring, emotion regulation, enhancement of self-esteem, problem-solving, and coping with sudden crises [23]. The e-MICHI app consisted of 6 modules containing psychoeducational texts, exercises, quizzes, and a total of 27 video clips (Table 2). Participants were encouraged to complete each module within 1 week. In addition to the modules, e-MICHI included other features such as a mood diary and an in-app messenger. The messenger enabled communication between participant and therapist via text message. Once a participant had completed a module, the therapist received an automatic notification via messenger (eg, “I completed lesson 1!”) and subsequently contacted the participant to address any queries or challenges related to the module. Participants could contact their therapist via messenger at any time if they had difficulty understanding the content of the modules or if they experienced a decrease in depressive symptoms. The app was hosted by Zone 35, a digital solutions provider [24].

**Table 2.** Detailed summary of e-MICHI modules included in the feasibility trial. Each module’s title, focus area, and corresponding therapeutic techniques are outlined.

	Content and psychoeducation	Exercises	Homework
1: “Knowledge of depression”	· Symptoms· Suicidality assessment	· Note personal takeaway^a^· Three quiz questions^a^	· Mood diary^b^
2: “Feelings”	· Depression causation· Mood improvement	· Personal disease model· Select pleasant activities· Identify strengths· Mind journey exercise	· Feedback on strengths· Pleasant activities· Mood diary^b^ (including activities)· Feedback on strengths
3: “Influence on feelings”	· Positive self-esteem· Thoughts-behavior-feelings link	· Note compliments· Record thoughts, behaviors, or feelings in situations	· Mood diary^b^ (behavior, thoughts, or feelings)· Practice complimenting
4: “Change thoughts”	· Crisis management· Cognition restructuring	· Note advice, link strategies to problems· Differentiate thoughts	· Personal emergency plan· Reflect on strategies with a therapist
5: “Support and health”	· Problem-solving skills	· Problem-solving exercise	· Appoint a “person of trust”· Share learned strategies
6: Refresher	· Quiz-based repetition	· Quiz on learned content	· No specific homework

a Repeats for lessons 1‐5.

b 7 days, 3 times per day.

Face-to-face contact was mandatory at baseline and after 3 and 6 weeks of using the app. Additional contact with the therapist was possible via messenger, phone, or as face-to-face contact, depending on the participant’s needs. Every contact was recorded in a protocol.

As suicidality is a common symptom of depression, especially in adolescents, the first module included information about suicidal thoughts and how to seek immediate help at the hospital in case of acute suicidality. Additionally, when consent was obtained, information was provided about procedures for serious suicidal behavior and suicidal behavior was clarified as part of the diagnostic process (see “Ethical Considerations”).

### Measures

Numerous assessments were made at baseline (T1), 3 weeks (T2), and 6 weeks (T3), as well as at the 3-month follow-up (T4). A full list is provided in Table 2. The following estimates have been used as a basis for the assessment of feasibility: adherence was assessed by recording the number of modules completed and the number of messages sent by participants to their therapist via the in-app messenger. Therapists documented their weekly interactions with the patient, including any additional contacts initiated by the patient and conducted between the patient and therapist (via phone, video call, or in-person) as well as adherence, among other things. An adherence score was created, based on the therapists’ weekly study documentation. For this purpose, 4 categories were used to rate each module, ranging from 0 (“little or no engagement”) to 3 (“excellent engagement,” meaning successful lesson completion, including the accomplishment of the everyday task). This rating was conducted by 2 independent raters; initially, each rater provided a rating per module, followed by reaching a consensus together, and then averaging over all evaluable modules.

Patient satisfaction was measured using a self-report questionnaire developed by the authors of the SPARX study [25], which was subsequently modified and translated for the purposes of this study. The questionnaire contained 37 quantitative and 5 qualitative items, which addressed various markers of user experience. The following section will focus exclusively on the items that were evaluated in the context of this paper. For an overall evaluation of the app the item “How would you rate the app overall on a scale of 1‐10?” was asked (1=bad and 10=excellent). The impact of the app was measured by rating the usefulness of 7 antidepressant strategies addressed in the app using a 5-point Likert scale ranging from 0 (“very useless”) to 4 (“very useful”). Transferability to daily life was measured by rating the frequency of use of 6 strategies on a 5-point Likert scale ranging from 0 (“never”) to 4 (“nearly every day”). Total scores for the scales “usefulness of the strategies” and “frequency of use of the strategies” were then calculated by summing the individual items within each scale.

Therapists’ overall satisfaction with the app was assessed using the item “How satisfied are you overall with the MICHI app?” on a scale from 1=not satisfied at all to 10=very satisfied. This item was part of a more extensive survey of therapists regarding their experiences with the app. A thorough evaluation of the therapists’ perspectives on the app will be presented elsewhere [26].

Trends of efficacy were measured by changes in BDI-II [17], CDRS-R [18], and Clinical Global Impressions-Severity Scale [19] scores. The cutoff value of 29 in the BDI-II rating at T1 was used to classify the severity of depressive symptoms as mild or moderate versus severe [17].

Adverse events (AE) were coded according to MedDRA (Medical Dictionary for Regulatory Activities) terminology. Detailed information collected for each AE included a description of the event, duration, whether the AE was serious, intensity, relationship to trial treatment, action taken, and clinical outcome. Summary tables presented the number of participants observed with AEs by MedDRA System Organ Class and Preferred Term, along with the corresponding percentages. Additional subcategories were based on event intensity and relationship to trial treatment.

### Ethical Considerations

The institutional review board of the Medical Faculty of the University of Rostock approved this study (registration number: A 2020‐0093). Before enrollment, participants and parents were verbally informed about the objective and methodology of this study and provided with separate written information documents. Written consent was then obtained from both parents and participants. Participants were informed that they could withdraw from the study at any time without negative consequences for their treatment. Participants aged 18 years did not require parental consent. Participants were not financially compensated for their participation in this study, as they were allowed to take part in an innovative new treatment program.

Suicidality was assessed at baseline and in the first module of the e-MICHI app. Participants were queried about whether they experience thoughts of death. They were informed that if they do have thoughts of death, their primary therapist would be notified via messenger and would reach out to them.

All data were deidentified for storage and subsequent evaluation, ensuring participant confidentiality throughout the research process.

### Procedures Against Bias

To reduce bias, independent raters, namely psychology students and research psychologists, assessed inclusion criteria and outcome measures, like the CDRS-R [18]. The raters underwent training in the administration of the diagnostic interviews, but were not acquainted with the participants’ psychiatric and psychotherapeutic histories. Furthermore, the assessment of adherence scores was conducted independently by 2 authors (LL and AS). Discrepancies between their findings were discussed and resolved.

### Data Analysis

Feasibility metrics were assessed by analyzing their central tendency and dispersion. Additionally, their association with the severity of depression was examined using appropriate statistical tests such as Pearson correlation coefficients, Mann-Whitney *U* tests, and 2-sample *t* tests.

Concerning trends of efficacy, 1-factor ANOVAs were conducted with 3 repeated measurements of the dependent variable (BDI-II and CDRS-R scores) within 1 sample, provided the prerequisite conditions were met. If the necessary conditions were not met, a nonparametric method was used for statistical analysis. Previous research has shown that using DHIs to treat depression in children and teenagers has mild to moderate positive effects [27]. As a result, we expected a moderate effect size of Cohen *f*=0.25 for this investigation. With a priori of 28 participants in total and a 5% probability of type I error, as well as a power of 0.8 and a 20% probability of type II error, the sample size was required to observe significant changes in the sample as per the protocol. The G*Power (Heinrich-Heine-Universität Düsseldorf) tool was used to carry out the sample size calculation [28]. Considering an attrition rate of approximately one-third than that was reported in previous trials involving smartphone app interventions for depressive symptoms [29], it was deemed as a suitable anticipated dropout rate for the investigation, ending up with a total of 36 enrolled participants. This sample size enabled us to explore the impact of covariates and associations between different variables. Furthermore, violations beyond the per-protocol sample of 28 participants could be recorded.

The main analysis was conducted with the per-protocol group, consisting of all participants from the intention-to-treat population who did not violate the study protocol, by failing to meet all inclusion criteria, meeting at least 1 exclusion criterion or being noncompliant to therapy.

Subgroup analyses were performed to identify potential variances among predictive factors, such as adherence, comorbid disorders, and treatment expectancy. The statistical test interpretation was exploratory, except for the trends of efficacy outcomes for which this study was powered.

## Results

### Participants and Dropout

**Figure 2.**
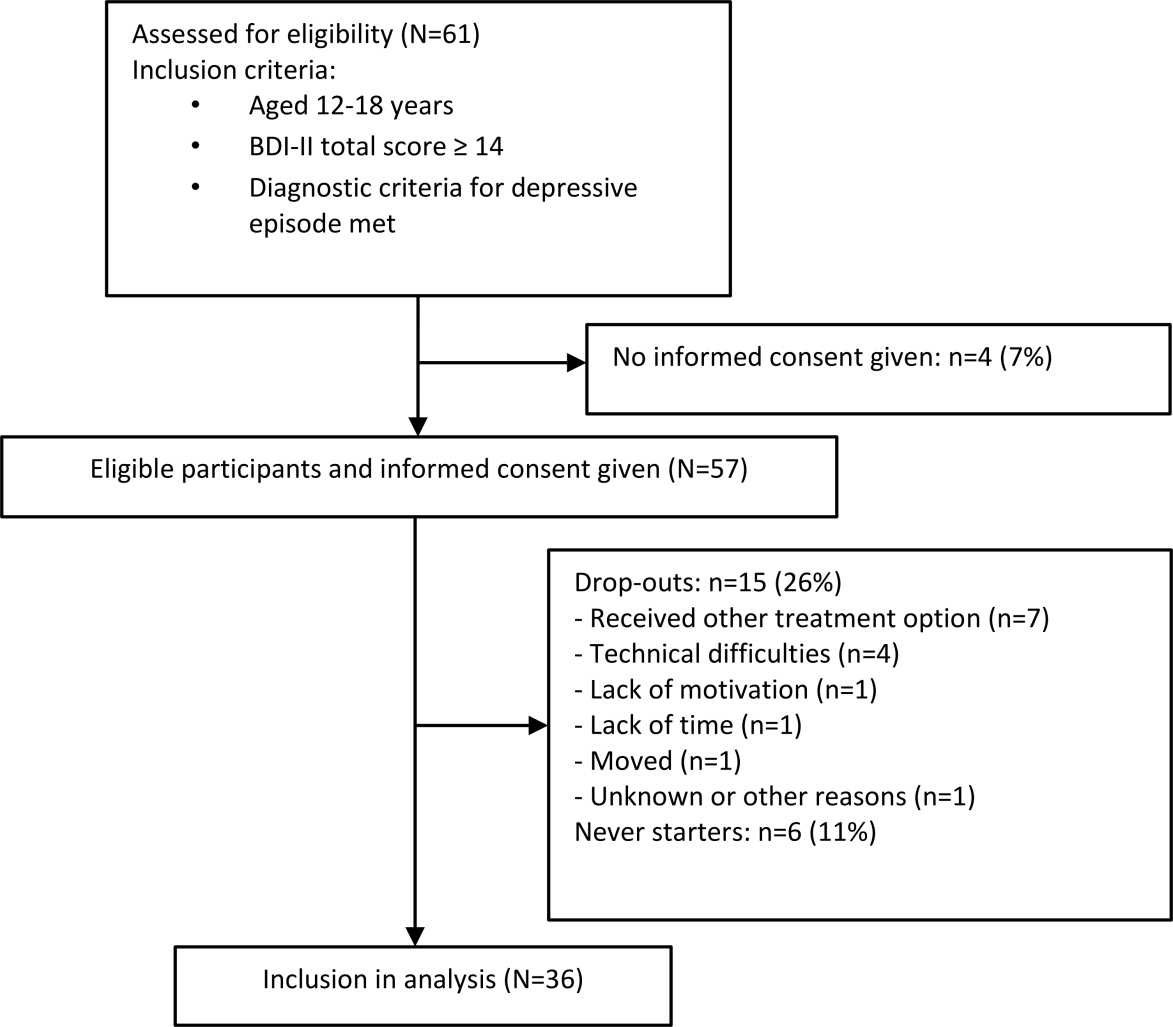
Participant flowchart depicting the recruitment, eligibility screening, and retention rate in the feasibility trial integrating the e-MICHI mental health app into a blended treatment of adolescent depression. BDI-II: Beck Depression Inventory-II.

A total of 61 participants were assessed for eligibility in this study. While informed consent was obtained from 57 individuals, 6 (11%) participants did not commence with the program. A further 15 (26%) participants dropped out (Figure 2). Dropouts were defined as participants who completed at least 1 lesson within the app but stopped using it for a variety of reasons, such as becoming unreachable for further contact, starting external outpatient psychotherapy, or being admitted to a day clinic or inpatient facility. For demographic data on the participants, please refer to Table 3. There was no significant difference in age or severity of depressive symptoms at T1 between the dropout group and the included participants. Further dropout analysis was conducted using a chi-square test to examine differences by study center, gender, school type, education, treatment expectancy, and previous psychiatric or psychotherapeutic experience. The results show no significant differences between dropouts and other participants on these variables. Thus, dropouts were deemed irrelevant for further analyses.

**Table 3.** Baseline demographic and clinical characteristics of participants (N=36) in the feasibility trial, including age, gender, distribution across outpatient centers, baseline depression severity, educational backgrounds, and prior treatment experience.

Characteristic			Values
Age, mean (SD)			
	Years		15 (1)
	Months		6 (7)
Age in years, n (%)			
	12		1 (3)
	13		7 (19)
	14		6 (17)
	15		10 (28)
	16		3 (8)
	17		8 (22)
	18		1 (3)
Gender, n (%)			
	Female		29 (83)
	Male		4 (11)
	Diverse		2 (6)
Center, n (%)			
	Neuruppin		17 (47)
	Rostock		19 (53)
Severity of depressive symptoms, n (%)			
	BDI-II<29		6 (17)
	BDI-II≥29		29 (83)
Education, n (%)			
	Grammar school		16 (46)
	Middle school		8 (23)
	Comprehensive school		4 (11)
	Vocational school		2 (6)
	Orientation stage		1 (3)
	Not attending school		4 (11)
Previous psychiatric or psychotherapy experience, mean (SD)			
	Years		1 (2)
	Months		8 (0)
Previous psychiatric or psychotherapy experience, n (%)			
	No previous experience		9 (26)
	≤1 year treatment		15 (43)
	>1 year treatment		11 (31)

### Adherence and Overall Satisfaction

While 50% (n=17) of participants successfully completed all modules, 74% (n=25) of participants completed at least the first 5 modules covering all therapeutic content (Table 4). Regarding patient communication via the app’s messaging feature, an average of 23 (SD 22.1) messages were sent per participant, with a high variance. According to the adherence score, almost 80% (n=25) of all participants showed good or excellent adherence (Table 4). On average, less than 1 additional contact initiated by the patient was documented by the therapist per week (mean 0.7, SD 0.31). There was no statistically significant difference in adherence score and messaging activity between participants with severe depressive symptoms and mild or moderate depressive symptoms (Table 4) and there was no correlation between the number of messages sent by participants and the number of lessons completed (*r*=.33, *P*=.06).

**Table 4.** Feasibility measures, including completion rates for app modules, adherence to the intervention, engagement via messages, additional contacts, and relationships between feasibility outcomes and depression severity.

Variables	Values	*P* value
Modules completed (N=34), mean (SD)	5.03 (1.27)	N/A^a^
Modules completed, n (%)		N/A
1	34 (100)	
2	34 (100)	
3	31 (91)	
4	30 (88)	
5	25 (74)	
6	17 (50)	
Adherence score (N=32), n (%)		N/A
0 (no or little engagement)	0 (0)	
1 (moderate engagement)	7 (22)	
2 (good engagement)	10 (31)	
3 (excellent engagement)	15 (47)	
Messages sent (N=34), mean (SD)	23 (22.1)	
Additional contact per week, mean (SD)	0.7 (0.31)	
Severity of depression, *t* test *(df)*		
Perceived usefulness	0.732 (25)	.47
Strategy implementation	0.808 (24)	.43
Severity of depression, Mann-Whitney *U*		
Modules complete	56.5	.76
Messages sent	63	.73
Patient app rating	40	.1

a N/A: not applicable.

Further analysis also showed no correlation between adherence score and therapy expectation (*r*=.17, *P*=.34), the overall satisfaction with the app (*r*=–.07, *P*=.73), and changes in depressive symptoms, as measured by both BDI-II (*r*=.18, *P*=.40) and CDRS-R (*r*=−.24, *P*=.19) scales. There was no statistically significant difference between the adherence scores of the group with previous psychiatric or psychotherapeutic experience and the group with no previous experience (*t*_30_=−1.22, *P*=.23).

Participants rated their overall satisfaction with the intervention as 7.3 on average (SD 1.2). Therapists indicated similar rates of overall satisfaction (mean 7.3, SD 1.1). Qualitative feedback from the therapists indicated that they particularly appreciated the inclusion of a mood diary due to its potential to monitor and reflect on emotional change.

### Impact of the App and Transferability to Daily Life

A detailed analysis on the perceived usefulness of the therapeutic techniques addressed in the e-MICHI app is presented in Table 5. In general, a high rate of participants indicated using therapeutic techniques in everyday life, with variance between different techniques (Table 6).

**Table 5.** Participant feedback on the perceived usefulness of various therapeutic techniques addressed in the e-MICHI app based on self-reported relevance to managing depression symptoms (N=27).

	Very useless or useless, n (%)	Unsure, n (%)	Very useful or useful, n (%)
Psychoeducation	1 (4)	6 (22)	20 (74)
Behavioral change or activation	7 (26)	5 (19)	15 (56)
Identification of negative thoughts^a^	3 (11)	12 (44)	12 (44)
Changing negative thinking^a^	6 (22)	10 (37)	11 (41)
Problem solving	5 (19)	9 (33)	13 (48)
Feel better about myself^a^	10 (37)	6 (22)	11 (41)
Emotion regulation	6 (22)	9 (33)	12 (44)

a As part of cognitive restructuring.

**Table 6.** Participant-reported frequency of practicing therapeutic techniques addressed in the e-MICHI app at the third assessment point (T3), reflecting the integration of these strategies into participants’ daily routines.

	N	Never, n (%)	A few times, n (%)	About once per week, n (%)	Several times per week, n (%)	Nearly every day, n (%)
Paid attention to basic needs	27	1 (4)	6 (22)	7 (26)	6 (22)	7 (26)
Spent time for activities that improve mood	27	1 (4)	3 (11)	7 (26)	11 (41)	5 (19)
Applied techniques of problem-solving	27	6 (22)	7 (26)	4 (15)	7 (26)	3 (11)
Identified positive things about yourself	27	2 (7)	6 (22)	9 (33)	7 (26)	3 (11)
Recognized negative automatic thoughts	27	3 (11)	6 (22)	6 (22)	9 (33)	3 (11)
Tried to change negative thoughts	26	2 (8)	7 (27)	6 (23)	9 (35)	2 (8)

A positive correlation was found between changes in BDI-II scores, reflecting a perceived decrease in depressive symptoms, and both the perceived overall usefulness of the techniques (*r*=.54, *P*=.004) and their total frequency of use (*r*=.62, *P*=.001). There was also a significant positive correlation between participants’ overall satisfaction with the app and the perceived usefulness of the techniques (*r*=.64, *P*<.001), as well as the frequency of their implementation (*r*=.70, *P*<.001).

Based on the severity of depressive symptoms, the analysis revealed no significant differences in the perceived utility of the techniques or their implementation frequency (see Table 4).

A more detailed investigation was carried out to explore whether the perceived effectiveness of e-MICHI in “tackling problems or issues causing difficulty” was related to the severity of depression. Using a Mann-Whitney *U* test, no significant difference in the evaluation of the item’s usefulness between participants with lower BDI-II scores and those with higher BDI-II scores was found (*U*=52.00, *Z*=−0.34, *P*=.76).

### Trends of Efficacy of e-MICHI

Depressive symptoms decreased significantly during the blended therapy, regardless of the measure used (BDI-II, CDRS-R, and Clinical Global Impressions-Severity Scale scores), and remained stable at follow-up 3 months after using the app (Table 7).

**Table 7.** Efficacy measures evaluating changes in self-reported depression severity, clinician-rated changes in depressive symptoms, and severity of illness from baseline (T1) to follow-up (T4) in the feasibility trial of e-MICHI.

Variables		Values
BDI-II^a^ (N=21)		
	T1, mean (SD)	37.71 (10.77)
	T2, mean (SD)	32.52 (12.89)
	T3, mean (SD)	30.62 (13.21)
	T4, mean (SD)	30.95 (15.38)
	*F* test (*df*)	4.10 (3, 60)
	*P* value	.01
CDRS-R^b^ (N=27)		
	T1, mean (SD)	58.93 (11.51)
	T3, mean (SD)	48.37 (15.28)
	T4, mean (SD)	42.48 (16.61)
	*F* test (*df*)	16.39 (2, 52)
	*P* value	<.001
CGI-S^c^ (N=29)		
	T1, mean (SD)	4.48 (0.79)
	T2, mean (SD)	3.97 (0.87)
	T3, mean (SD)	3.48 (0.95)
	T4, mean (SD)	3.38 (1.32)
	Chi-square (*df*)	33.45 (3)
	*P* value	<.001

a BDI-II: Beck Depression Inventory-II.

b CDRS-R: Children’s Depression Rating Scale-Revised (clinician rating).

c CGI-S: Clinical Global Impressions-Severity Scale.

### About AEs

There were 6 AEs of nonsuicidal self-injury, 1 of which was a serious adverse event (SAE) that required inpatient admission for crisis intervention. Thoughts of death were reported on 12 occasions, but there were no indications of acute suicidality. Another AE involved an outpatient procedure for a nonrelated medical condition. None of the 7 AEs, including the SAE, were deemed related to the intervention and this study’s therapy continued unchanged.

Of the 7 dropouts who received other treatment options, 3 were inpatients and 2 were day patients (planned partial hospitalization), both categories coded as SAE. However, these SAEs were not considered to be related to the intervention. Additionally, there were 2 dropouts where study treatment was discontinued due to other outpatient treatment options.

## Discussion

### Principal Findings

This study aimed to investigate the feasibility of the DHI e-MICHI as a blended intervention to treat mild, moderate, and severe depression among adolescents. We found that adolescents adhered well to the intervention, indicated general satisfaction as well as a reduction in depressive symptoms from both patient and therapist perspectives. The use of the app was feasible in a population of adolescents with clinically significant depression and significant effects were observed in child and adolescent psychiatric outpatients. Improvement of depressive symptoms measured at the end of the intervention remained stable at the 3-month follow-up.

Crucial points in e-mental health applications are adherence and transferability to everyday practice [13]. We observed high adherence rates, which are comparable to other blended interventions [30] and substantially higher than unguided e-health applications [31,32]. In this study, participant satisfaction and reported transfer to daily practice were high. This differs from results of other studies, such as the SPARX-R program [12], which reported lower perceived usefulness and transfers in daily practice in adolescents excluded from mainstream education. A high transfer of psychotherapeutic exercises and skills into everyday life could be one of the most important benefits of using apps in psychotherapeutic practice. Apps could intensify therapy and with more individualized feedback, for example, based on ecological momentary assessment or generated by AI, a tailored personalized psychotherapeutic intervention could be provided.

Concerning adherence, a substantial proportion of participants, about 3-quarters, completed the core modules of e-MICHI. Reasons for high adherence may include aesthetic design [33], relevant content [34], or the perceived immediacy of support through the app’s messenger as stated by participants. These findings align with the principles highlighted by Domhardt et al [8]. Adolescents also reported applying the strategies they learned from the app in their daily lives, a promising sign of real-life transfer, even among those who rated their depressive symptoms as severe. The high adherence may be influenced by the fact that our study population consisted of adolescents seeking psychiatric-psychotherapeutic treatment who were clinically significantly depressed and impaired by their disorder. Interestingly, our findings indicate no significant differences in adherence between participants with long or short-term and no prior therapeutic experience. This highlights the possibility that even individuals with prior experience in therapy may derive benefits from e-MICHI suggesting the intervention could serve as a valuable adjunct to traditional therapeutic approaches, enhancing treatment outcomes and promoting sustained engagement across diverse patient populations. Additionally, the absence of an existing therapeutic relationship for adequate adherence would not limit the use of DHIs, for example, to bridge the gap for face-to-face psychotherapy or as a primary intervention. Notably, adolescents appreciated the close contact with therapists provided by the messaging function, highlighting the added benefit in blended approaches [35]. DHIs may therefore be more beneficial when embedded in conventional care settings.

The acceptance of the app is evidenced by the high satisfaction ratings from participants and therapists alike. The high acceptance rate, especially among adolescents, may be partly due to their general openness to digital interventions [6]. In combination with the therapeutic relationship and the facilitated way of communicating directly with the therapist via the app, e-MICHI, or DHIs in general, seem to be an added value in psychiatric-psychotherapeutic treatment for adolescents.

The inclusion of a mood diary, as appreciated by the therapists, could enhance therapeutic engagement and outcomes due to its potential to track and reflect on emotional changes [36]. This consensus, together with the high level of satisfaction, supports the usefulness of the app as a complementary tool in psychotherapy and its potential to strengthen the therapeutic alliance, as also shown by Tremain et al [37].

In our study, additional contacts beyond the planned 3 sessions were rare, indicating efficient management of patient care with reduced direct contact frequency compared to our clinical experience, where patients in outpatient settings are typically seen every 1 to 2 weeks. Additionally, compared to treatment as usual, messenger contacts helped provide feedback on mood diary entries and suggested steps for e-MICHI daily task implementation.

This finding aligns with the objective of providing more accessible care [38] as it can reduce both waiting times to start psychotherapy and the need to travel long distances to receive care. It must be noted, that technical problems and difficulties with devices or limited network coverage or access to free Wi-Fi may be barriers to the use of therapeutic apps, especially for adolescents. In this study, 4/57 (7%) of originally included participants dropped out due to technical problems. Access to free Wi-Fi seems to be essential for adolescents to be able to use the app in their living environments. Ways to use the app offline would be desirable to make it accessible to even more patients.

Regarding trends of efficacy, there was a noticeable decrease in depressive symptoms during the intervention, as reported by both participants and external evaluators. This significant improvement highlights the potential of e-MICHI in the treatment of depression, warranting further validation through randomized controlled trials (RCTs) to more robustly establish its clinical efficacy.

Given the sample, which included participants with severe depression, drop out due to referral to other psychiatric treatment services was not surprising. The advantage of the blended approach is evident in the case of a patient who was admitted for crisis intervention and was able to resume the intervention after discharge providing continuity of care and additional therapist support, consequently increasing adherence.

Regarding safety, this study presents an optimistic perspective on the e-MICHI blended CBT intervention for adolescents with depression, as evidenced by a small number of unrelated AEs and no suicide attempts. Comparatively, Rasing et al [39] observed 2 suicide attempts in a similar blended CBT intervention, but found no significant differences in AEs between face-to-face and blended treatment conditions. A recent review indicated no increased risk of suicidality with blended treatment for adolescents with depression [40]. Concerning self-harm, Fleming et al [41] reported a comparable number of 2 incidents in a similar sample size, neither of which were related to the intervention. The absence of suicide attempts during the e-MICHI intervention, along with the findings from Rasing et al [39] and Fleming et al [41], suggests that blended interventions may not substantially increase the risk of severe AEs, such as suicide attempts and self-harm. Nonetheless, Domhardt et al [8] emphasize the need for continued vigilance and monitoring in blended mental health interventions, especially for high-risk populations.

Contrasting the results of e-MICHI with similar blended interventions, e-MICHI allowed participants to use their own devices, incorporating beneficial strategies such as text messaging for homework reminders and self-monitoring between sessions [42], and included participants with severe depressive symptoms, addressing the criticism regarding the exclusion of such cases in previous research [43]. As noted, we found no significant difference in adherence rates in participants with severe depressive symptoms. While this is consistent with previous findings [44], this result may also be due to the small sample size.

Comparing e-MICHI to unguided internet-based CBT [40] highlights the potential benefits of a blended approach. The higher adherence rates and satisfaction scores suggest that integrating therapist support into an e-health platform may enhance the perceived usefulness of these interventions, as well as how well adolescents are able to apply the therapeutic strategies they learn to their daily lives.

### Limitations

The non-RCT design of this study limits the generalizability of our findings on efficacy. As a feasibility study we wanted to detect trends of efficacy; the trial was not designed for efficacy testing. An RCT will be the next step in generating evidence for the e-MICHI intervention. Most of this study’s population had a history of psychotherapy. Therefore, our participants might be more familiar with therapeutic contents than adolescents who use such a therapeutic app as a first-time psychotherapeutic intervention. The adherence scoring method faced challenges due to several therapy documentations missing or lacking sufficient information for rating. Given the small sample size, it is possible that these factors could have influenced the findings. For the development of therapeutic interventions according to precision medicine, it would be of interest to know about precise times of use of specific contents (eg, in the morning, evening, etc), direct effects on symptoms (eg, mood), etc. Unfortunately, due to technical and privacy restrictions, we were unable to evaluate this data.

### Conclusion

Due to its easy application, the e-MICHI intervention seems to be a promising tool to enhance face-to-face treatment of depression in adolescents. This study identified areas for further development of the app based on user feedback and technical issues, to improve its effectiveness and user experience in the future. It contributes to filling the existing evidence gap on the feasibility and efficacy of e-therapy interventions via apps for adolescents with depression in the German health care system. According to the results of this study, the potential benefits of blended therapy for depression by intensifying therapy and facilitating patient-therapist communication are evident. As digital interventions continue to evolve, further research is imperative to address challenges, for example, accommodating a broad range of individuals, crisis intervention, and high dropout rates, ensuring that such platforms can effectively serve as viable therapeutic options for adolescents with depression.

## Abbreviations

AE: adverse event
BDI-II: Beck Depression Inventory-II
CBT: cognitive behavioral therapy
CDRS-R: Children’s Depression Rating Scale-Revised
DHI: digital health intervention
*ICD-10*: *International Statistical Classification of Diseases and Related Health Problems, 10th Revision*
MDD: major depressive disorder
MedDRA: Medical Dictionary for Regulatory Activities
MICHI: Manualized Intervention to Cope With Depressive Symptoms, Help Strengthen Resources, and Improve Emotion Regulation
RCT: randomized controlled trial
SAE: serious adverse event
